# Vivostat Platelet-Rich Fibrin^®^ for Complicated or Chronic Wounds—A Pilot Study

**DOI:** 10.3390/biomedicines8080276

**Published:** 2020-08-06

**Authors:** Andreas Bayer, Gesa Höntsch, Mark Kaschwich, Annika Dell, Markus Siggelkow, Rouven Berndt, Rene Rusch, Jürgen Harder, Regine Gläser, Jochen Cremer

**Affiliations:** 1 Institute of Anatomy, Kiel University, Olshausenstr. 40, 24098 Kiel, Germany; 2 Department of Heart and Vascular Surgery, University Hospital of Schleswig-Holstein, Campus Kiel, Arnold-Heller-Str. 3, Haus C, 24105 Kiel, Germany; gesahoentsch@web.de (G.H.); rouven.berndt@uksh.de (R.B.); rene.rusch@uksh.de (R.R.); jochen.cremer@uksh.de (J.C.); 3 Department of Surgery, University Hospital of Schleswig-Holstein, Campus Lübeck, Ratzeburger Allee 160, 23538 Lübeck, Germany; mark.kaschwich@uksh.de (M.K.); Annika.dell@gmail.com (A.D.); 4 Department of Vascular and Thoracic Surgery, Imland Clinic Rendsburg, Lilienstraße 20–28, 24768 Rendsburg, Germany; Markus.Siggelkow@imland.de; 5 Department of Dermatology, Kiel University, Rosalind-Franklin-Str. 9, 24105 Kiel, Germany; jharder@dermatology.uni-kiel.de (J.H.); rglaeser@dermatology.uni-kiel.de (R.G.)

**Keywords:** chronic wound, complicated wound, Vivostat platelet-rich fibrin (PRF), individualized wound therapy

## Abstract

Vivostat Platelet-Rich Fibrin^®^ (PRF) is an autologous platelet concentrate used for the local treatment of chronic or complicated wounds. Still, its application for this indication is not evidence-based. Therefore, we performed this monocentric retrospective pilot study investigating the clinical outcome of a local treatment of chronic or complicated wounds in 35 patients (23 male, 12 female, mean age 68.7 years) treated with Vivostat PRF^®^. This study population is the largest among published studies analyzing the clinical efficacy of Vivostat PRF^®^ on chronic wounds so far. Using the perpendicular method we divided the wounds into three sizes (<10, 10–30, and >30 cm^2^). The clinical efficacy of the Vivostat PRF treatment was the primary endpoint and was divided into three groups of increasing degrees of wound improvement: (1) no improvement of the wound (wound area was not reduced > 10% under Vivostat PRF^®^ treatment), (2) improvement of the wound (reduced area > 10% under Vivostat PRF^®^ treatment) and (3) complete epithelialization (wounds that were completely re-epithelialized after Vivostat PRF^®^ treatment). We included patients’ diagnosis and concomitant diseases (peripheral arterial occlusive disease (PAOD)), chronic venous insufficiency (CVI)), diabetic foot syndrome (DFS)) in our data analysis in order to investigate their potential impact on the wound healing capacity of Vivostat PRF^®^. Our results show that in the entire study population, 13 out of 35 (37.1%) patients experienced wound improvement and 14 out of 35 (40%) patients showed complete epithelialization of their wound under Vivostat PRF^®^ treatment. In summary, 77.1% of the treated patients benefited from the Vivostat PRF^®^ therapy. These positive wound healing effects were all observed within the first three to six Vivostat PRF^®^ applications. Subgroup analyses showed that Vivostat PRF^®^ appeared to be more efficient in patients without CVI in comparison to patients with CVI (*p* = 0.02). Moreover, Vivostat PRF^®^ treatment seems to be particularly efficient in PAOD-related wounds with a reduced crural arterial blood supply (*p* = 0.01). Additionally, we performed an experimental human in vivo study on ten male students where we artificially generated bilateral gluteal wounds and analyzed the influence of the Vivostat PRF^®^ treatment on the expression of two genes (human beta Defensin-2, ((hBD-2) and human beta-Defensin-3 (hBD-3)) in keratinocytes of resected wound specimens that are induced during wound healing. Interestingly, this analysis revealed that only seven of out ten individuals showed a relevant hBD-2 and hBD-3 gene induction after Vivostat PRF^®^ treatment. This led to the novel “key-lock-hypothesis”. With the goal of an individualized precision medicine approach with optimized wound treatment strategies in the future, this is an important observation that demands further experimental and clinical studies.

## 1. Introduction

Chronic wounds are not only a great burden for affected patients, but also impose an immense financial strain on the healthcare system. The prevalence and incidence of chronic wounds continue to rise. In Germany, one million people suffered from chronic wounds in 2012 [1]. Patients’ quality of life is significantly reduced due to physical, psychological and social restrictions caused by the chronic wound [2,3,4]. Costs to the healthcare system for the treatment of these patients comprise of around 9600 euros per patient per year [5]. In conclusion, the therapy of chronic or complicated wounds is often lengthy, expensive and frustrating for both the patient and the therapist. One emerging therapeutic option for the treatment of chronic or complicated wounds is the topical application of autologous thrombocyte concentrate lysates. These contain a magnitude of chemokines, cytokines and growth hormones which are used to stimulate tissue regeneration in general [6,7], and to support the complex process of wound healing in particular [8,9,10,11].

Despite many reports of positive clinical effects of the usage of thrombocyte concentrate products for the treatment of chronic or complicated wounds [12,13,14,15,16,17], their use for this indication is still not evidence-based [13]. Therefore, we performed this pilot study to analyze the clinical efficacy of the Vivostat PRF^®^ treatment on complicated or chronic wounds in patients suffering from peripheral arterial occlusive disease (PAOD) with concomitant chronic venous insufficiency (CVI) and diabetic foot syndrome (DFS) that are typically treated in a department for vascular surgery.

## 2. Material and Methods

### 2.1. Definition of A Chronic Wound

According to the current German S3 guideline “Local therapy of chronic wounds with the risks factors CVI, PAOD and/or diabetes mellitus”, a wound is defined as being chronic when it displays a loss of skin integrity and one or more underlying structures with no healing within eight weeks. The average age of the chronic wounds treated with Vivostat PRF^®^ (Vivostat A/S, 3450 Lillerød, Denmark), in this study was 34 months (range between 2.5–134 months).

### 2.2. Definition of A Complicated Wound

We defined wounds as being complicated when they showed a loss of skin integrity and one or more underlying structures. This could be caused by a postoperative wound healing disturbance, in which wound healing is substantially delayed, due to infection, for example. In contrast to chronic wounds, complicated wounds have not yet existed for eight weeks.

### 2.3. Clinical Study Population

We analyzed the data of 35 patients whose wounds were treated with Vivostat PRF^®^ at the Department of Heart- and Vascular Surgery of the University Clinic of Schleswig-Holstein, Campus Kiel. All patients received unsuccessful professional standard wound therapy prior to the Vivostat PRF^®^ treatment and could therefore be regarded as an internal control population. All 35 patients provided written informed consent to take part into this study. We included 23 men and 12 women in our analysis. These patients were aged between 40 and 99 years, and the average age was 68.71 years. Diagnoses and concomitant diseases of the analyzed patients are presented in Table 1.

### 2.4. Vivostat PRF^®^ Treatment

Before application of Vivostat PRF^®^, the wounds were cleaned with sterile NaCl 0.9% or polyhexanide (SERASEPT^®^, SERAG-WIESSNER GmbH, Naila, Germany) and debrided when indicated. Preparation and application of Vivostat PRF^®^ were performed as indicated by Vivostat A/S. After Vivostat PRF^®^ application, we used a Polyurethane (PU) foam dressing (Biatain^®^ adhesive, Coloplast GmbH, Hamburg, Germany) for wound coverage that was fixed on the wound for at least five days. Vivostat PRF^®^ treatments were repeated according to the clinical course of wound healing.

### 2.5. Analyses of the Clinical Efficacy of the Vivostat PRF^®^ Treatment

We investigated the clinical efficacy of a topical Vivostat PRF^®^ treatment by measuring the wound area before and after treatment with the perpendicular method [18,19]. The perpendicular method is recommended by the German Society for Wound Healing and Wound Therapy as the method of choice to investigate a possible influence of a certain “tool” on the course of wound healing.

Wounds were divided into three groups according to their initial size:

(a) wounds with a size <10 cm^2^
(b) wounds with a size >10 cm^2^ and <30 cm^2^
(c) wounds with a size >30 cm^2^.

To investigate the wound healing capacity of Vivostat PRF^®^, we divided treated patients into three groups:

(a) “no improvement of the wound” in patients where the wound size was reduced <10% under Vivostat PRF^®^ treatment
(b) “improvement of the wound” in patients with a reduced size >10% under Vivostat PRF^®^ treatment
(c) “complete epithelialization” in patients with completely re-epithelialized wounds after Vivostat PRF^®^ treatment.

The primary endpoint of this study was the reduction in wound area measured and categorized as described above.

### 2.6. Statistics

All data were initially pseudonymized. Statistical analysis were performed by SPSS, version 25 using the chi-square test (likelihood ratio). All variables that showed a tendency (*p*-value < 0.15) were included into a logistic regression model. The odds ratio (OR) was given with a confidence interval of 95%. In all statistical tests, a *p*-value ˂ 0.05 was regarded as being significant.

### 2.7. Experimental Study Population

**Figure 1:**
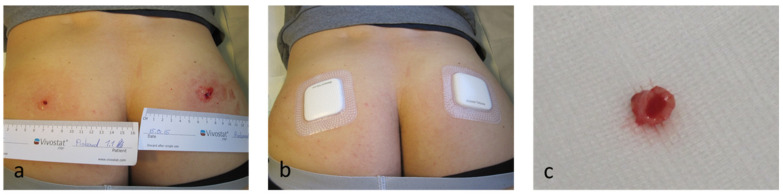
In our experimental human in vivo study we generated bilateral gluteal wounds with sterile 4 mm biopsy punches on 10 male students (**a**). Left gluteal wounds were treated with NaCL as control, right gluteal wounds were treated with Vivostat Platelet-Rich Fibrin (PRF^®^) on day 0 and day 5. Treated wounds were covered with sterile PU-foam dressings (**b**). On day 10 we resected bilateral gluteal wound specimens with 6 mm biopsy punches (**c**).

With the approval of the ethics committee of the Medical Faculty of the Christian-Albrechts-University of Kiel (AZ A115/13), we performed a human in vivo study on 10 male students aged between 19 and 28 years. Exclusion criteria were nicotine abuse, diabetes mellitus or other diseases known to impair wound healing. In this study we generated bilateral gluteal wounds with a sterile 4 mm biopsy punch in every individual (see Figure 1a). Left gluteal wounds were treated with sterile NaCL as controls, right gluteal wounds were treated with Vivostat PRF^®^ on day 0. Bilateral wound coverage was performed afterwards as described above for the clinical study population (see Figure 1b). On the fifth day, this treatment was repeated: left gluteal wounds were treated with sterile NaCl, while right gluteal wounds were treated with Vivostat PRF^®^. On day 10 after wound generation, we resected bilateral gluteal wounds from all 10 participants with 6 mm biopsy punches and performed primary surgical wound closure under sterile conditions. From the resected specimen (see Figure 1c), we performed RNA-isolation, reverse transcription, cDNA-synthesis and analyses of several genes that are assumed to influence the complex wound healing process as described before [20,21].

## 3. Results in the Clinical Study Population

### 3.1. Entire Study Population

**Figure 2:**
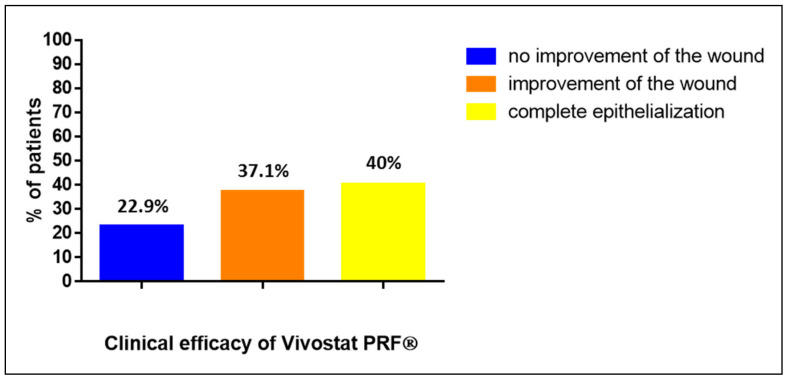
Clinical efficacy of the Vivostat PRF^®^ treatment in the entire study population (*n* = 35).

In the entire study population (*n* = 35), we included 29 patients with chronic or complicated wounds on their lower legs due to PAOD, CVI and diabetic foot syndrome. Six patients had their wounds elsewhere: two patients with chronic or complicated wounds after sternotomy, two patients with chronic or complicated wounds after laparotomy and two patients with chronic or complicated inguinal wounds after vascular surgery. After analyzing these 35 patients, we observed that 8 patients (22.9%) treated with Vivostat PRF^®^ showed no improvement in their wound, 13 patients (37.1%) showed an improvement in their wound and 14 patients (40%) developed complete reepithelization of their treated wounds (see Figure 2).

### 3.2. Number of Vivostat PRF^®^ Treatments

**Figure 3:**
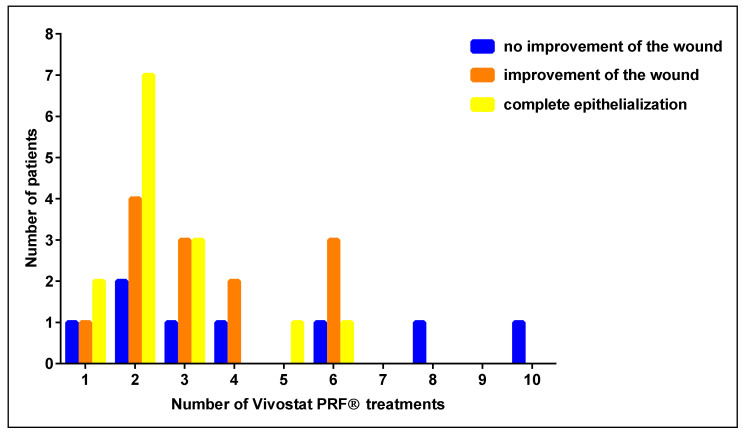
Number and clinical efficacy of Vivostat PRF^®^ treatments.

In the entire study population (*n* = 35), we analyzed whether there could be a correlation between the number and the clinical success of the Vivostat PRF^®^ treatment. All 35 patients were treated with Vivostat PRF^®^ between one and ten times. In this analysis, we observed that if patients benefit from the Vivostat PRF^®^ treatment, it is evident after the first three applications in the vast majority of treated patients (see Figure 3.).

### 3.3. Impact of PAOD on the Clinical Efficacy of Vivostat PRF^®^

**Figure 4:**
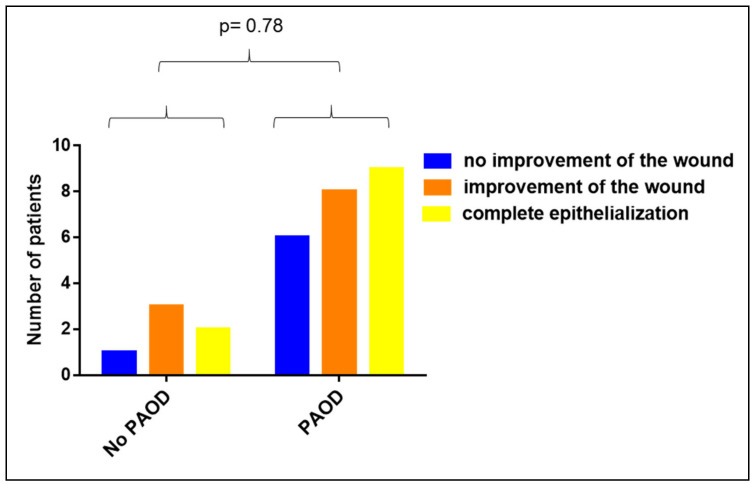
Impact of peripheral arterial occlusive disease (PAOD) on the clinical efficacy of Vivostat PRF^®^ (*p* = 0.78).

We analyzed the impact of peripheral arterial occlusive disease (PAOD) on the clinical efficacy of the Vivostat PRF^®^ wound therapy. Comparing patients with and without PAOD revealed that a PAOD had no impact on the wound healing capacity of Vivostat PRF^®^ (*p* = 0.78, see Figure 4).

**Figure 5:**
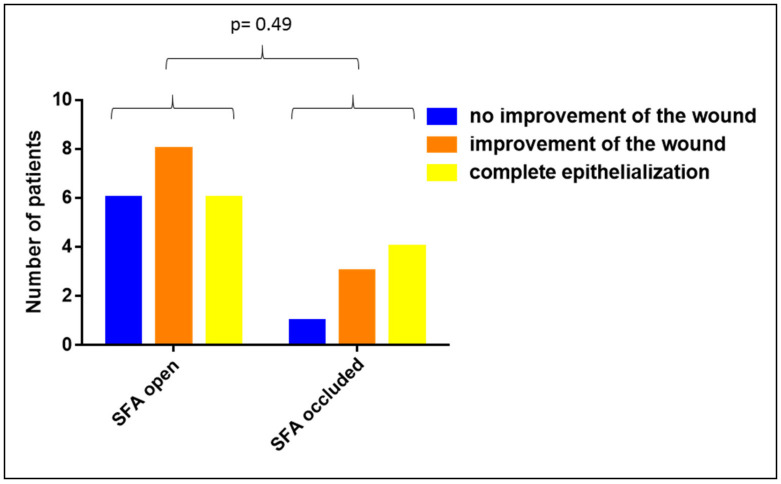
Impact of SFA patency on the clinical efficacy of Vivostat PRF^®^.

To investigate whether the clinical efficacy of Vivostat PRF^®^ in patients with a PAOD of the thigh differs from patients with a PAOD of the calf, we analyzed the impact of the patency of the superficial femoral artery (SFA) and the crural arteries on the wound healing capacity of Vivostat PRF^®^. Comparing patients with an open SFA with patients with an occluded SFA showed no relevant impact of the SFA patency on the wound healing efficacy of Vivostat PRF (*p* = 0.49, see Figure 5).

**Figure 6:**
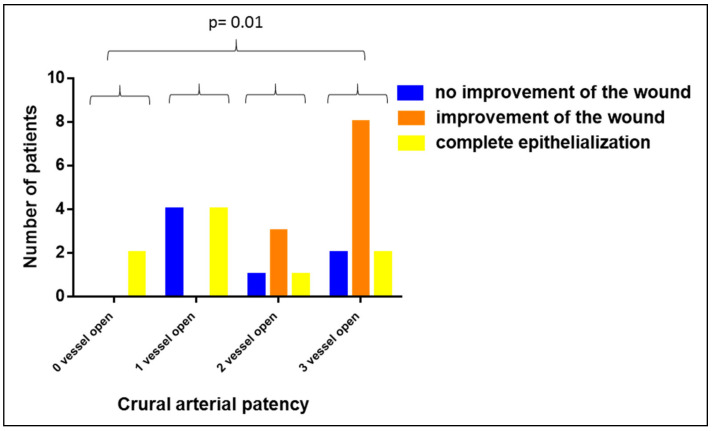
Impact of the crural arterial patency on the wound healing capacity of Vivostat PRF^®^.

In contrast, when comparing patients with 0, 1, 2 and 3 open crural arteries, we observed that the worse the crural arterial blood supply of the lower leg and the foot were, the more efficient the Vivostat PRF^®^ therapy was (*p* = 0.01, see Figure 6). In this analysis, we included the A. dorsalis pedis, the A. tibialis posterior and the A. fibularis as the three vessels of interest.

### 3.4. Impact of CVI on the Clinical Efficacy of Vivostat PRF^®^

**Figure 7:**
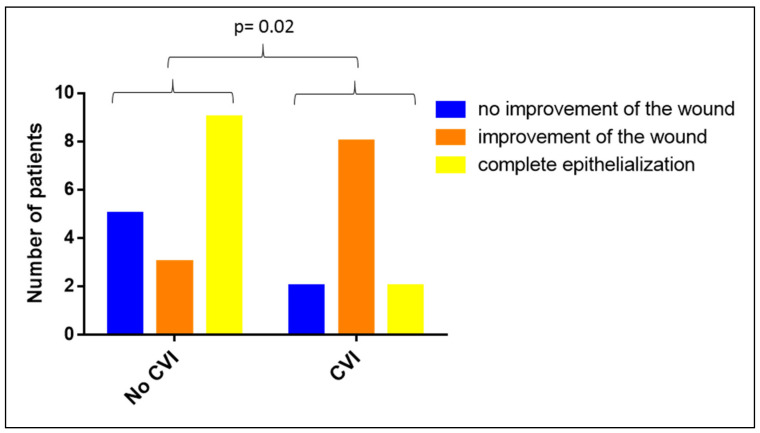
Impact of chronic venous insufficiency (CVI) on the clinical efficacy of Vivostat PRF^®^.

Included in this analysis are all patients with wounds on their lower extremities, regardless of whether they also had a PAOD or diabetic foot syndrome (*n* = 29). Our analysis of the impact of a CVI on the clinical efficiency of Vivostat PRF^®^ revealed a negative impact of an existing CVI on the clinical efficiency of a Vivostat PRF^®^ wound therapy (*p* = 0.02, see Figure 7), indicating that patients without a CVI benefit more from a Vivostat PRF^®^ treatment than patients with a CVI.

### 3.5. Impact of Diabetic Foot Syndrome (DFS) on the Clinical Efficacy of Vivostat PRF^®^

**Figure 8:**
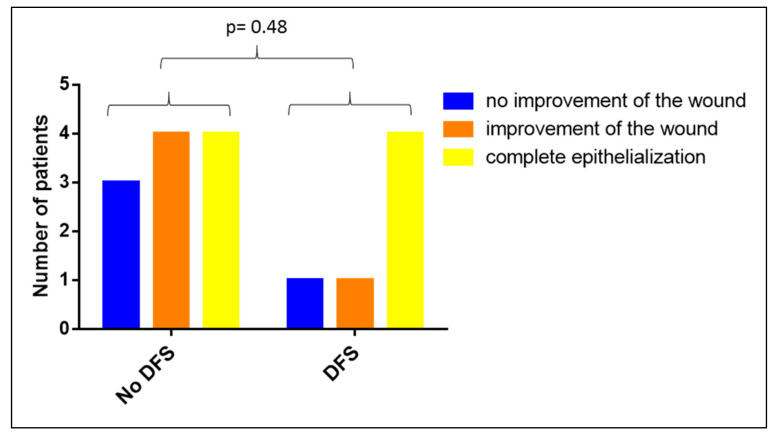
Impact of diabetic food syndrome (DFS) on the clinical efficacy of Vivostat PRF^®^.

In this analysis, we included all patients with wounds on their lower extremities and with diabetes or diabetic foot syndrome regardless of whether they also had a PAOD or a CVI (*n* = 17). Comparing patients with and without a DFS showed that diabetic foot syndrome had no impact on the clinical efficiency of the Vivostat PRF^®^ treatment (*p* = 0.48, see Figure 8).

### 3.6. Impact of the Duration of the Wound-Existence on the Clinical Efficacy of Vivostat PRF^®^

**Figure 9:**
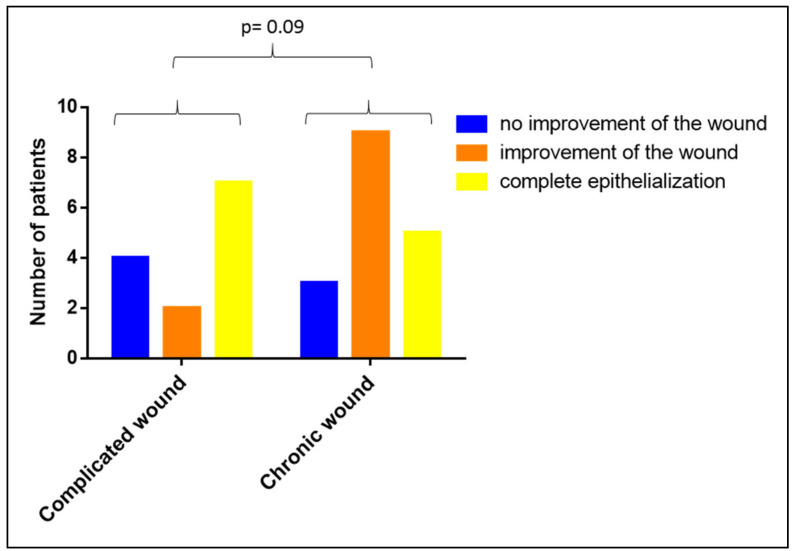
Impact of the duration of the wound-existence on the clinical efficacy of Vivostat PRF^®^.

We were also interested in analyzing whether the duration of the wound’s existence has an impact on the clinical efficiency of the Vivostat PRF^®^ treatment. When comparing chronic with complicated wounds, we observed no difference in the clinical efficiency of the Vivostat PRF^®^ therapy between complicated and chronic wounds (*p* = 0.09, see Figure 9).

### 3.7. Impact of the Initial Wound Size on the Clinical Efficacy of Vivostat PRF^®^

**Figure 10:**
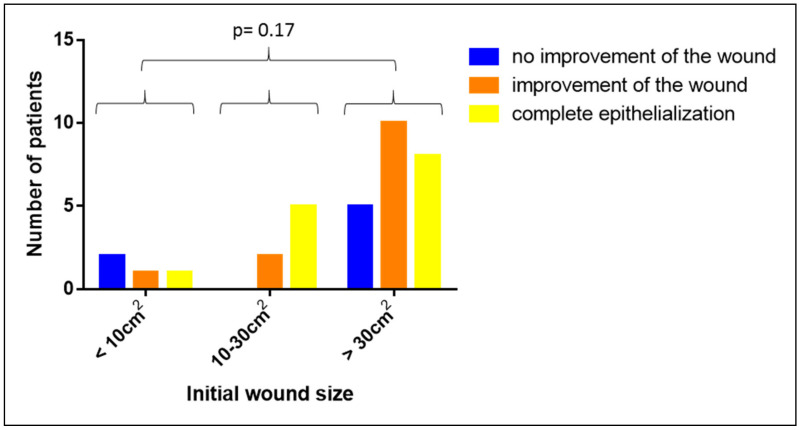
Impact of the initial wound size on the clinical efficacy of Vivostat PRF^®^.

Our analysis on the potential impact of the initial wound size on the clinical efficacy of the Vivostat PRF^®^ treatment revealed no significant influence (*p* = 0.17, see Figure 10). This indicates that Vivostat PRF^®^ is equally potent in small as well as in large wounds.

## 4. Results in the Experimental Study Population

In the resected specimen of the artificially generated bilateral gluteal wounds of all 10 male study participants, we analyzed the influence of the performed Vivostat PRF^®^ treatment on human-beta defensin-2 (hBD-2) and human beta defensin-3 (hBD-3) gene expression, two genes that are induced during the complex wound healing process.

**Figure 11:**
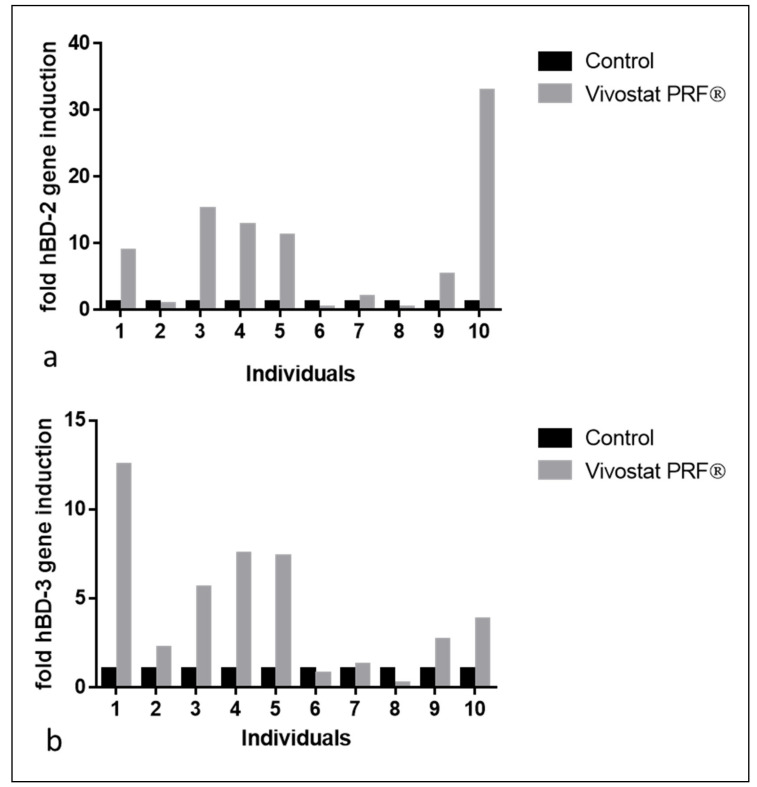
Seven out of ten individuals in our experimental Vivostat PRF^®^ study showed a relevant hBD-2 (**a**) and hBD-3 (**b**) gene induction in the resected wound specimens 10 days after Vivostat PRF^®^ treatment.

Analyses of hBD-2 and hBD-3 gene expression in the entire experimental study population revealed significant hBD-2 and hBD-3 gene induction in Vivostat PRF^®^ treated wounds as described before [20,21]. Separate analyses of the individual Vivostat PRF-mediated hBD-2 and hBD-3 gene induction in all 10 treated study participants revealed that 7 out of 10 individuals (individuals 1, 3, 4, 5, 7, 9, 10) showed hBD-2 gene induction after Vivostat PRF^®^ treatment. Individuals 1, 2, 3, 4, 5, 9 and 10 showed hBD-3 gene induction after Vivostat PRF^®^ treatment (see Figure 11). This indicates that only individuals 1, 3, 4, 5, 9 and 10 (60% of treated individuals) reacted with a relevant hBD-2 or hBD-3 gene induction after Vivostat PRF^®^ treatment.

## 5. Discussion

Due to their supposed tissue regeneration capacity [7,8], autologous thrombocyte products are used with increasing frequency for many indications in several medical disciplines [22,23,24,25,26]. In the context of chronic or complicated wounds, they have already been used for the stimulation of wound healing [14,27,28]. Despite some reports of their positive clinical wound healing capacity, the clinical efficacy of a wound treatment with thrombocyte concentrate products is still discussed controversially [13]. One might speculate on the underlying reasons. In general, the comparability of studies on the wound healing capacities of thrombocyte concentrate products are limited due to diverse options to evaluate an assumed clinical wound healing effect (e.g., wound volume, wound area, volume of wound exudate, frequency of dressing exchange, local pain in the wound area). Furthermore, it has been shown that the concentration of growth factors and other factors (e.g., fibrin) included in the product influence the clinical efficacy of a certain thrombocyte product used for tissue regeneration or wound healing [29,30,31]. This impairs the comparability of studies dealing with this issue.

As Vivostat PRF^®^ is proven to contain a magnitude of concentrated growth factors, e.g., transforming growth factor-β1 (TGF-ß1), platelet-derived growth factor (PDGF), vascular endothelial growth factor (VEGF), basic fibroblast growth factor (BFGF) [11] and fibrin, we used this autologous thrombocyte product for the stimulation of wound healing in chronic or complicated wounds since 2009 in our department. The contained fibrin component is especially important as it fixes the growth factors on the wound and prevents admission into the dressing. To our mind, this is mandatory for successful wound therapy with thrombocyte concentrate products, as the growth factors should act on the wound over a timescale of days. This seems to be of high importance, as in vitro experiments have revealed that the influence of thrombocyte concentrate products on gene expression in human keratinocytes depends on the duration of exposure [20]. Furthermore, it has already been shown that Vivostat PRF^®^ induced several antimicrobial peptides, e.g., human beta-Defensin-2 (hBD-2), human beta-Defensin-3 (hBD-3) and psoriasin [20,21,32] and accelerated epidermal differentiation [33] in artificially generated wounds after 10 days of Vivostat PRF^®^ treatment. For these reasons, we regard Vivostat PRF^®^ as an autologous thrombocyte concentrate lysate suitable for wound healing therapy.

In our analyses, we observed that the majority of treated patients (77.1%) benefited from the Vivostat PRF^®^ therapy. This is in line with data from Steenvorde et al. [12]. Interestingly, we observed that wounds improved mostly within the first three to six Vivostat PRF^®^ applications. Comparable effects have been described by O’Connel by using a platelet-rich fibrin matrix membrane [34]. Therefore, we believe that continued Vivostat PRF^®^ therapy without any positive wound healing effects after these three to six Vivostat PRF^®^ applications seems not to be indicated as having a positive effect on the wound. In a clinical context, this is very significant information, as many desperate chronic wound patients demand to continue their Vivostat PRF^®^ treatment, even if no positive wound healing effect is visible after three to six Vivostat PRF^®^ applications.

Due to the limited number of included patients, our statistical analyses concerning the impact of underlying diseases as PAOD, CVI and diabetic food syndrome has to be interpreted critically. Because the crural arteries should be the “last meadow” and the body has only a few opportunities to compensate for a reduced crural arterial blood supply, our observation that PAOD wound patients with a reduced crural arterial blood supply do especially benefit from Vivostat PRF^®^ therapy could be interpreted as a consequence of the enormous regenerative capacity of Vivostat PRF^®^.

Although it has been described that PRF could be clinically efficient for the therapy of chronic venous leg ulcers in the majority of patients [34] we observed that Vivostat PRF^®^ might not be comparably efficient in patients with venous leg ulcers. One might speculate that different underlying pathophysiological processes might explain these contrasting findings.

In former studies, thrombocyte products have been proven to be especially efficient to promote wound healing in patients with diabetic food syndrome (DFS) [14,35] and have been proposed as a new standard therapy for this indication [6]. Interestingly, we have not detected a relevant impact on the clinical wound healing capacity of Vivostat PRF^®^ in the context of a diabetic food syndrome. However, due to the limited number of patients included in our study who suffer from DFS, further studies in this context are required.

On the basis of our clinical data, it seems that the initial wound size and the duration of the wound existence do not have an impact on the clinical wound healing capacity of Vivostat PRF^®^.

The comparatively small number of patients included in our study naturally limits the validity of the data presented. This is a well-known problem of clinical studies with small patient numbers in general and in wound-related investigations in particular. For this reason, our study should also be interpreted first of all as a pilot study [36,37]. Of course, the data presented here have to be verified on larger numbers of patients with chronic or complicated wounds. Therefore, a prospective randomized multicenter trial or registry would be helpful.

The results of our experimental study on the effect of a repeated Vivostat PRF^®^ treatment on hBD-2 and hBD-3 gene induction in 10 male participants reveal that only six to seven out of ten treated individuals showed relevant gene induction of hBD-2 and hBD-3 in their keratinocytes of the resected specimen after repeated Vivostat PRF^®^ treatment. Although it is conceivable that individuals with chronic wounds could respond differently to the Vivostat PRF^®^ treatment from how young and healthy students do, we have determined that only 60–70% of analyzed individuals could be regarded as “responders” to the performed Vivostat PRF^®^ treatment in our experimental study population. Three to four out of these 10 individuals showed no relevant hBD-2 and hBD-3 gene induction and had to be regarded as “non-responders” of the performed Vivostat PRF^®^ treatment. Interestingly, the proportion of “responders” in our clinical and experimental study population is comparable. We can only speculate on underlying reasons for this observation. Maybe not all human individuals are sensitive to wound therapy with autologous thrombocyte products because they are lacking specific extra- or intracellular receptor and/or signal transduction molecules. This “key-lock-hypothesis” would also be in line with our observation discussed above that the clinical efficacy of a Vivostat PRF^®^ treatment is normally observed after the first three applications, implying that individuals that benefit from this special wound therapy do so immediately. Moreover, this “key-lock-hypothesis” would argue for an individualized precision medicine approach where chronic wound patients will be tested on their individual ability to respond to wound therapy with autologous thrombocyte products before the therapy is started. Generation of 2D or 3D skin models with keratinocytes isolated from autologous hair follicles and subsequent stimulation with autologous thrombocyte products may offer such a pre-treatment option [38]. Such an approach would be comparable to patients with breast cancer where the estrogen receptor status is analyzed before starting anti-estrogen therapy. As underlying signal transduction pathways of autologous thrombocyte products in keratinocytes and other cells involved in the complex wound healing process are not yet sufficiently investigated, this could be one focus of future studies.

## 6. Conclusions

This is the largest pilot study showing that the majority of patients with chronic or complicated wounds benefit from a topical wound therapy with Vivostat PRF^®^. Of course, on the one hand the interpretation of the data presented is limited due to the relative low number of patients analyzed; on the other hand, these results reflect our clinical observations after treatment of around 200 patients over the last ten years with Vivostat PRF^®^, supporting the concept that Vivostat PRF^®^ is a very potent tool for the treatment of chronic or complicated wounds. This seems to be particularly true for patients with a PAOD based on an impaired crural arterial blood supply. Wound size does not influence the clinical efficacy of a Vivostat PRF^®^ treatment. A continued Vivostat PRF^®^ wound therapy without any positive clinical wound healing effects after three to six applications seems not to be promising. Of course, our novel “key-lock-hypothesis” has to be confirmed in larger trials.

Future studies should focus on the individual ability of chronic wound patients to respond to autologous thrombocyte concentrate products before starting this therapy. With the aim of an individualized precision medicine, this approach would optimize this special wound therapy in the future.

## Figures and Tables

**Table 1:** Diagnoses and concomitant diseases of analyzed patients.

Diagnoses and Concomitant Diseases of Analyzed Patients	*n* (%)
Peripheral arterial occlusive disease (PAOD)	29 (82.85%)
Chronic venous insufficiency (CVI)	12 (34.29%)
Diabetes mellitus	17 (48.57%)
Diabetic foot syndrome (DFS)	6 (17.14%)
Obesity	8 (22.86%)
Arterial hypertension	27 (77.14%)
Renal insufficiency	17 (48.57%)
Hyperlipidemia	15 (42.86%)
Nicotine abuse	11 (31.43%)
